# Impact of Global Transcriptional Silencing on Cell Cycle Regulation and Chromosome Segregation in Early Mammalian Embryos

**DOI:** 10.3390/ijms22169073

**Published:** 2021-08-23

**Authors:** Martin Anger, Lenka Radonova, Adela Horakova, Diana Sekach, Marketa Charousova

**Affiliations:** Central European Institute of Technology, Department of Genetics and Reproduction, Veterinary Research Institute, 621 00 Brno, Czech Republic; radonova@vri.cz (L.R.); horakova@vri.cz (A.H.); sekach@vri.cz (D.S.); charousova@vri.cz (M.C.)

**Keywords:** oocyte, embryo, cell cycle, translation, transcriptional repression

## Abstract

The onset of an early development is, in mammals, characterized by profound changes of multiple aspects of cellular morphology and behavior. These are including, but not limited to, fertilization and the merging of parental genomes with a subsequent transition from the meiotic into the mitotic cycle, followed by global changes of chromatin epigenetic modifications, a gradual decrease in cell size and the initiation of gene expression from the newly formed embryonic genome. Some of these important, and sometimes also dramatic, changes are executed within the period during which the gene transcription is globally silenced or not progressed, and the regulation of most cellular activities, including those mentioned above, relies on controlled translation. It is known that the blastomeres within an early embryo are prone to chromosome segregation errors, which might, when affecting a significant proportion of a cell within the embryo, compromise its further development. In this review, we discuss how the absence of transcription affects the transition from the oocyte to the embryo and what impact global transcriptional silencing might have on the basic cell cycle and chromosome segregation controlling mechanisms.

## 1. Peculiar Life of Mammalian Oocytes and Early Embryos

After entering meiosis during early intrauterine development, mammalian oocytes arrest in prophase of the first meiotic division and remain stored in the cortex of the ovary, enclosed by follicular cells [1]. The arrest continues for a prolonged time—in some species, for decades—until follicles are sequentially recruited for growth by hormonal stimulation after puberty. Upon stimulation, the follicles rapidly increase their size during this period, which, in mice, takes approximately three weeks, and oocytes simultaneously produce a glycoprotein coat called the *zona pellucida*. With completion of the growth period and the synthesis of a sufficient amount of stockpiled mRNAs and proteins, oocytes become ready for the resumption of meiosis, which is characterized by two meiotic divisions without DNA replication in between. After the first meiotic division, during which one set of homologous chromosomes is segregated into the first polar body, oocytes arrest again, this time in the metaphase of the second meiotic division. This second meiotic arrest is achieved by the activity of the Cytostatic Factor (CSF) and lasts for hours [2,3]. Fertilization, which is a very complex process requiring specific receptors on both the oocyte and sperm, triggers the release of metaphase II arrest, which leads to the activation of the Anaphase-Promoting Complex (APC/C), destruction of cyclin B1 and anaphase entry [4]. During fertilization, the sperm brings to the oocyte several important components, namely, another haploid set of chromosomes and phospholipase C zeta (PLCζ) [5]. PLCζ is responsible for the release of the CSF block, and in some species, the sperm also provides the centriole [6]. After the completion of the S phase, the maternal and paternal pronuclei in the newly formed embryo move into position close to each other, but in the mammals studied so far, they never fuse. In the zygote, the paternal and maternal chromosomes are initially separated during the prophase on two simultaneously assembling spindles. Both spindles eventually merge together, forming a single spindle with both parental sets of chromosomes [7]. The proper arrangement of parental DNA in both pronuclei prior to the division is a very delicate process, which might, if not properly executed, hinder the accurate segregation of chromosomes in the forthcoming anaphase [8]. Shortly after sperm penetration, the second polar body, containing a maternal set of sister chromatids, is extruded. The cleavage cycles, in which the size of the embryonic blastomeres halves during each division, continue until the first differentiation. This gives rise to the first two cell lineages, inner cell mass and trophoblast cells, which eventually become a proper embryo and placenta, respectively. From above, it is clear that female germ cells have a unique ability to arrest and resume the cell cycle after a prolonged time, and meiotic resumption subsequently initiates rapid and profound changes concerning every aspect of cell morphology and behavior. The extremely complex events of the completion of meiosis, fertilization and transition into an embryo are even more astonishing if we consider that they are mostly controlled by a regulated translation. In the following sections, we would like to discuss the recent progress in our understanding of the possible impact of global transcriptional arrest on regulation of the cell cycle and chromosome segregation in mammalian oocytes and early embryos.

## 2. The Onset of Transcriptional Silencing during Female Meiosis

The transcription ceases towards the completion of the growth period and finally fades away completely in fully grown germinal vesicle (GV) oocytes coincidentally with achieving meiotic competence [9]. Silencing of the transcription is further accompanied by changes of the chromatin configuration from non-surrounding nucleolus (NSN) with chromatin dispersed throughout the germinal vesicle, and into the surrounding nucleolus (SN) with the chromatin condensed and telomeres and centromeres clustered [10,11,12,13,14]. The absence of global transcription lasts until the awakening of the embryonic genome during the process called Zygotic Genome Activation (ZGA), which occurs after fertilization when the new embryo is formed [9,15,16], although a minor transcription appears in many species already shortly after fertilization, as will be discussed further. In addition to the absence of the global transcription, the oocytes, coincidentally with the resumption of meiosis, initiate the degradation of the maternal transcripts, which is then largely completed by ZGA [17,18,19,20]. The exact molecular mechanisms responsible for the transcriptional repression in mammalian oocytes are not yet fully understood.

## 3. Activation of Zygotic Genome in Embryos

The duration of the period without transcription, lasting from transcriptional silencing in full-grown oocytes until the major ZGA in embryos, varies between species (reviewed in references [9,15,21]). Similar to the repression of the transcription, the details concerning the molecular control over the resumption of the transcription after fertilization are not yet fully understood. Among the processes that have been so far linked to the onset of transcription from the newly formed embryonic genome are changes in the nucleocytoplasmic ratio, maternal clock associated with the cell cycle, chromatin remodeling and histone modifications [15,21,22]. From above, it seems that the onset of ZGA requires the orchestration of multiple processes, which were studied previously, but we do not know yet how they are interdependent. In this context, it is also important to mention recent discoveries concerning the chromatin organization in mouse zygotes, which seems to be different in comparison to the organization of chromatin domains in somatic cells. It is possible that such a specific arrangement of chromatin contributes to the lack of effective transcription in early embryos after fertilization [23,24,25].

The onset of transcription seems to be organized into two waves, and such an arrangement is conserved in many species [21,22]. In mice, the first wave of transcription was detected already in the zygote [26], whereas the major burst of transcription followed in the two-cell embryos stage [27,28]. In humans, the major wave of transcription occurs later than in mice, around the four-to-eight-cell stage of embryonic development [29]. However, a low level of transcription, specifically concerning the genes related to the protein transport and signaling, was also detected already in zygotes [17]. In cattle, representing another large mammalian species in which the onset of ZGA was studied, the timing of ZGA seems to be largely similar to humans, with a minor transcriptional activity in the zygotes and a large increase in transcription between the four-cell and eight-cell embryo stages [30,31,32]. Recent progress in the sensitivity of molecular biology methods allowed the detailed characterization of the nascent transcription from the embryonic genome during the first wave of transcription. This initial and low-level transcription was detected in multiple species already in the zygote phase. In mice, it was, however, shown that it is rather inefficient, and the produced mRNAs are poorly processed, which concerns specifically the splicing and polyadenylation [33]. It seems, therefore, that, in mammals, transcription ceases in oocytes, coincidentally reaching their full size and meiotic competence, and it reappears after the fertilization, depending on the species, from the second to fifth interphase. 

## 4. Transcription-Independent Regulation of Cell Cycle in Oocytes and Early Cleavage Embryos

**Figure 1:**
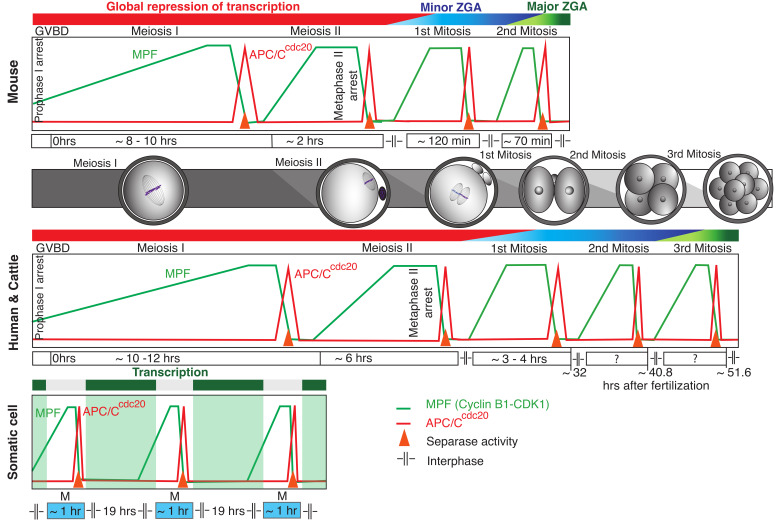
The differences in the utilization of transcription for cell cycle regulation between mouse, human and cattle oocytes and early embryos (upper and middle panel) and somatic cells (lower panel). The relative duration of the cell cycle stages and timing of the activity of MPF, APC/C and separase are indicated. Whereas, in oocytes, transcription is silenced in fully grown oocytes and then restarted during the minor and major waves of ZGA, somatic cells have the ability to replenish major cell cycle regulators by transcription during the interphase. The execution of cell cycles during early development is therefore fully dependent on the regulated translation of transcripts accumulated at the GV stage. In the case of proteins that are targeted during each anaphase, such as cyclin B, a controlled translation is crucial. Information from the following publications was used for the figure preparation: references [47,48,49,50,51,52,53,54].

In all known eukaryotic cells, the cell cycle is driven by the sequential activation of Cyclin-Dependent Kinases (CDKs), which phosphorylate a set of substrates specific for each cell cycle stage (reviewed in references [34,35]). The phosphorylation by CDKs is opposed by phosphatases, reverting the phosphorylation status of proteins, and together, they drive cells through the transitions between the cell cycle stages [36,37]. Equally important for accurate cell cycle progression is also the controlled proteolysis of specific protein substrates. This function is accomplished by a two-step mechanism consisting of the ligation of a target protein with ubiquitin during the first step, which is mediated by APC/C, and the subsequent proteolysis of the ubiquitinated protein at the proteasome [38,39]. The coordination of ubiquitination and the proteolysis of important cell cycle regulators ensure the unidirectionality of the cell cycle and, in multicellular organisms, provides another level of control over cell multiplication. In somatic cells, certain cell cycle regulators, such as cyclin B1, are targeted by APC/C and destroyed by proteolysis during each metaphase-to-anaphase transition, and their levels are then restored in the subsequent cell cycle by coupled transcription and translation [40,41,42,43]. Regulated transcription is therefore an essential part of the control of cell cycle progression. In oocytes and early cleavage embryos, however, the majority of the transcription is silenced, which requires these cells to employ compensatory mechanisms. Transcriptional silencing is also known from somatic cells, in which the condensation of chromosomes and dissolution of the nuclear membrane upon mitotic entry lead to the less frequent association of Polymerase II (Pol II) and transcription factors with chromosomes [44]. A recent work, however, showed that a low-level transcription can be detected even during mitosis [45]. In contrast to this, the fully grown mammalian oocytes that acquired the competence to successfully complete both meiotic divisions seemed to lack transcription completely [9,46]. The full repression of transcription, resuming in some species only after four or five cell divisions (from meiosis I to the eight-cell embryos stage), is unique and challenging in many aspects (Figure 1). In particular, because, during that time, oocytes engage in very complex events requiring the orchestration of multiple processes and demanding the expression of large cohorts of specific proteins. Two consecutive meiotic divisions, which perhaps are more complex than mitosis, fertilization, which involves the interactions of specific receptors and the fusion of two completely different cells, and the formation of a zygote with the parental genetic material merged are just some of them. Simultaneously, oocytes and embryos maintain cell cycle progression and control of the fidelity of chromosome segregation. It is obvious that the germ cells are well-adapted for this situation and employ several mechanisms that allow them to cope with the absence of transcription.

One of the mechanisms of adjusting to transcriptional repression and, at the same time, allowing faster cell cycle progression might be a simplification of a repertoire of molecules essential for executing cell cycle transitions in embryos. Gene deletion studies have demonstrated that the absence of many genes, which are considered to be essential for the cell cycle, arrest embryos only at the later stages of early development [54,55,56,57,58]. In the case of cyclins—for example, type *D cyclins*—their absence arrests the development as late as during E16.5 [59]; in the case of *E cyclins*, it is E11.5 [60,61]. On the other hand, it was demonstrated that the core cell cycle machinery in the cleavage mouse embryos requires *cyclin B1* [62]. When using *in vitro* developing *cyclin B1* null mouse embryos, it was shown that the absence of this cyclin arrests blastomeres at the G2/M phase of the four-cell embryos stage [63]. Equally important is cyclin A2, which in mice meiosis II is required for the fidelity of sister chromatid segregation [64] and arrests embryonic development upon its deletion after 5.5 days [65]. Contrary to the results of gene targeting, it was, however, shown that the polyadenylation-dependent expression of cyclin A2 in mouse zygotes seems to be required for ZGA [66]. In somatic cells, cyclin A2 is essential for the initiation of DNA replication, as well as for G2/M transition [67,68]. During DNA replication, cyclin A2 plays a role in the complex with CDK2 [67], as well as directly without an association with CDK [69]. It seems therefore unlikely that DNA replication in the embryo could be accomplished without *cyclin A2*, as suggested by gene deletion. Although it was shown that, in certain cell types, the role of cyclin A2 in DNA replication might be substituted by cyclin E complexes [70], it is not clear whether such redundancy would be possible in early embryos. In the case of CDKs, CDK1 seems to be the only CDK required until organogenesis, and CDK2, CDK3, CDK4 and CDK6 are dispensable during this period [71]. However, the deletion of *CDK1* allowed 16% of embryos to develop until 3.5 days post-fertilization [72], which indicates that the maternally provided stockpile of CDK1 runs out later than that of cyclin B1. This might be caused by a general abundance of CDKs to cyclins, as well as by the APC/C and proteasome targeting of cyclin B1 during each cell division, leading to a faster depletion of its mRNA. In general, a better understanding of the regulation of early cleavage cycles in mammalian embryos would perhaps require a combination of gene deletion with the simultaneous depletion of a specific mRNA. A combination of both approaches would show better which molecules are essential during this period and whether the simplification of cell cycle control, suggested by gene deletion studies, is the case. In somatic cells, the cell cycle control exhibits a high degree of redundancy [73]; however, without further studies, it is still not clear whether this redundancy is temporally lost in early embryos.

The main adaptation of oocytes and embryos to the absence of transcription is perhaps regulated translation [74,75]. The global transcriptional silencing and inability to resynthesize mRNAs encoding important cell cycle control genes is the main reason why these cells extensively utilize controlled translation. A well-known mechanism is regulated polyadenylation, although the translation of certain molecules—for example, CDK1/CDK2 activators from the RINGO/Speedy protein family—extensively studied in *Xenopus* oocytes is polyadenylation-independent [76,77]. Regulated polyadenylation is conserved between species and controls the expression of multiple proteins [78,79]. It was extensively studied in the case of cyclin B1, whose expression in the period of global transcriptional repression is achieved solely by controlled translation [80]. It requires a specific sequence in cyclin B1 mRNA 3′ UTR, which is recognized by the Cytoplasmic polyadenylation element-binding protein (CPEB) or Embryonic poly(A)-binding protein (EPAB) [81,82] and, also, by other factors, which leads to polyadenylation and the recruitment of cyclin B1 mRNA for translation (reviewed in reference [83]). Recently, it was shown that the translation of cyclin B1 in mouse oocytes is additionally controlled by different lengths of cyclin B1 3′ UTR [84]. Polyadenylation seems to be generally used in oocytes and early embryos to compensate for the absence of transcription, but in mammals, there is no comprehensive information on importance of this process for maintaining cell cycle regulators other than the cyclin B1.

## 5. The Control of Chromosome Separation in Oocytes and Early Embryos

Separation of the sister chromatids during the somatic cell cycle is facilitated by the removal of a protein complex called cohesin, which holds the sister chromatids together from the DNA replication [85]. In vertebrates, cohesin is removed by a two-step mechanism during which the majority of cohesin at the chromosome arms is removed by the prophase pathway at the onset of mitosis [86] and simultaneously with the displacement of Pol II from the chromosome arms [87], whereas cohesin, located in the vicinity of centromeres, holds together the sister chromatids until the anaphase, during which, it is cleaved by an enzyme called separase [88,89]. It seems that, in oocytes, cohesin is removed solely by separase during the anaphase, and the prophase pathway is absent [90]. A similar situation is perhaps in early embryos [6]. Besides the crucial role of separase in mitosis, this enzyme in somatic cells has other functions—for example, in the separation of centrioles [91,92]. Separase also has a specific role in oocytes besides cohesion cleavage, which is to cleave the kinetochore protein Meikin involved in Rec8 protection during meiosis I [93]. To our knowledge, there is no information about the regulation of separase mRNA or the protein levels during the somatic cell cycle, though more so in embryos; however, recent data from yeasts suggest that the translation of separase is not very efficient [94]. Although we do not have direct evidence, the requirement of separase activity during mitosis, as well as during the interphase, suggests that its expression might be stable throughout the cell cycle. It is also equally important to control the activity of this enzyme, since a precocious segregation of the sister chromatids would render cells aneuploid. The activation of separase during chromosome division must be linked to the proper assembly of the spindle apparatus and the attachment of all kinetochores to spindle microtubules. In somatic cells, separase activity is regulated by several redundant mechanisms, including phosphorylation by the cyclin B1/CDK1 complex and by binding to a specific inhibitor called securin [89]. Crucially, both inhibitory pathways are linked to the proper spindle assembly and correct kinetochore attachment by a pathway called the Spindle Assembly Checkpoint (SAC). Upon proper spindle assembly, the SAC is turned off, which leads to activation of the APC/C and polyubiquitination and proteasome degradation of cyclin B1 and securin [95]. Recently, it was shown that the activity of separase is additionally controlled by the complex of Mad2 and Sgo2 [96], but the significance of this regulation for early development remains to be elucidated. How separase expression is controlled during early development and whether the protein or mRNA or both are stabilized or maintained by other mechanisms—for example, by mRNA polyadenylation—is unknown. It is, however, clear, that the enzyme must be active during the anaphase. Of paramount is, however, how early embryonic blastomeres control separase activity in order to prevent aneuploidy caused by a precocious separation of the sister chromatids. The data from mouse early embryonic development suggests that a separase control by CDK1 phosphorylation is essential during the cleavage cycles [97]. However, securin also has an important role in the control of separase activity, at least in the two-cell embryos stage. It was shown that the deletion of the APC/C activator *CDC20* arrests mouse embryos at the two-cell metaphase stage with high cyclin B1 and securin protein levels [98]. The removal of securin, although with a significant delay, triggers chromosome segregation in this situation, providing the evidence that the securin also plays an important role in controlling separase activity in embryos.

## 6. The Control of Spindle Assembly in Oocytes and Early Embryos

Assembly of the spindle is, in somatic cells, controlled by a pathway known as the SAC, which critically depends on the kinetochores (reviewed in reference [99,100]). It seems, however, that the SAC in oocytes works differently than in somatic cells, and, for example, congression defects are tolerated in oocytes [101,102,103]. A possible explanation of this phenomenon in oocytes might be their size, which shifts the ratio between the volume of the cytoplasm and the signal from the kinetochores in favor of a larger cytoplasm in these cells [104]. Whether the SAC function is compromised in oocytes or early blastomeres due to their size is still an open question, since another study using oocytes [105] or a recent report using blastomeres of early embryos [106] found no link between the cell size and fidelity of SAC. Similar to oocytes, it is not clear whether the SAC in early embryonic blastomeres is fully functional. It was shown that, in response to spindle poison, the blastomeres of the two-cell embryos stage are capable of recruiting Mad2 to the kinetochores [98]. It was also shown that the inhibition of Mps1 by reversine significantly increased the frequency of aneuploidy from the two-to-four-cell embryo stages [107]. Other results, however, indicated that the SAC function might be compromised. For example, hyperploid blastomeres showing signs of DNA fragmentation and exhibiting significant delays in the duration of the cell cycle stages are capable of proliferating until the blastocyst stage [108]. Blastomeres of the mouse four-to-eight-cell embryos are unable to postpone the anaphase in the presence of misaligned chromosomes [106]. This indicates that the SAC not only in oocytes but, also, in early mouse embryos behaves differently, in comparison to somatic cells. It seems that the main difference might be its inability to postpone the anaphase in response to chromosome congression defects and lagging chromosomes. Additionally, in meiosis II, it is not clear whether SAC plays any role. These oocytes are arrested at metaphase II by the CSF, for which the activity of SAC is dispensable [109]. MII arrest lasts several hours, until fertilization or oocyte degradation, and it is conceivable that oocytes might be prone to chromosome attachment errors during this prolonged arrest. However, a recent study showed that oocytes in meiosis II are capable of correcting improper kinetochore attachments, even during the metaphase-to-anaphase transition [110], when the SAC should be already silenced. Chromosome segregation errors increase with maternal age, and in this regard, the precocious segregation of sister chromatids, detectable in meiosis II, plays a leading role in age-related aneuploidy [111]. If SAC was functional in meiosis II, such oocytes should have been eliminated because of the presence of single chromatids unable to establish a bipolar connection to the spindle. Gene knockout studies have shown that the disruption of *Mad1*, *Mad2*, *Bub1*, *Bub3* and *BubRI*, which are essential genes from the SAC pathway, is embryonically lethal as early as E4.5, and the embryos suffer from aneuploidy and apoptosis [112,113,114,115,116,117]. This argues for the importance of this pathway in mouse early embryos. However, we have no information about the stability of the maternal mRNAs or how the expression of the above genes is achieved before E4.5. Additionally, we only have limited information about the SAC in the early embryos of other mammalian species. In human preimplantation embryos, it was shown that mRNAs encoding Mad2 and Bub1 are expressed at a very low level during preimplantation development, and their concentrations within blastomeres increase only after hatching [118]. It was shown in *Xenopus* oocytes that multiple mRNAs encoding essential SAC proteins, including Mad1, Mad2 and BubR1, are activated for cytoplasmic polyadenylation element (CPE)-dependent translation in a manner similar to cyclin B1 [119]. We have, however, no evidence whether such a mechanism is utilized either to control the translation of SAC components in mammalian oocytes or the embryos.

## 7. Conclusions

It is known that chromosome segregation in mammalian oocytes and embryos is highly error-prone in comparison to somatic cells, and at the same time, aneuploidy is the most frequent cause of termination of development (reviewed in reference [120,121]). The factors contributing to aneuploidy were extensively studied in mouse and human oocytes, and it seems that prolonged meiosis I arrest, an unusual mechanism of the spindle assembly and a large volume of cytoplasm causing SAC to ignore misaligned chromosomes are the main reasons behind the high frequency of aneuploidy in mammalian oocytes and early embryonic blastomeres in general. Due to the specific life cycle of oocytes, maternal age further contributes to the increase of aneuploidy in humans. Early mammalian embryos share an extremely high frequency of aneuploidy with oocytes. The data from clinical centers showed that more than 70% of the embryos grown *in vitro* are affected by aneuploidy [122], and in mouse *in vivo* embryos, the frequency of mosaic aneuploidy was found to be similar to human embryos [108]. It is conceivable that the error-sensing and -correcting mechanisms, such as cell cycle checkpoints, were adjusted throughout evolution to the somatic cells rather than to the germ cells. Chromosome segregation errors in somatic cells might cause aneuploidy and, subsequently, cancer in multicellular organisms [123], whereas the same errors in oocytes or in early embryos before implantation lead to the termination of development. There is, however, also a possibility that complex adaptations of transcriptional repression during early development when the cell cycle and control mechanisms of chromosome segregation rely on the regulated translation of maternally provided mRNAs are not optimal for the fidelity of chromosome segregation. In order to obtain a better understanding of how the cell cycle control mechanisms are impacted during early development, we need to obtain more experimental data, which will address the levels of the important regulators during the early cleavage cycles, especially in humans and other species, with postponed ZGA. We also need to study how the control pathways—for example, SAC—are assembled during early development and whether they retain full functionality, as shown in somatic cells. Using advanced microscopy and sequencing techniques, this should be addressed at the level of individual blastomeres in developing embryos, because aneuploidy affects, in most cases, only a small cohort of cells within an embryo.

## Data Availability

Not applicable.
